# In vitro antibacterial and antibiotic modifying activity of crude extract, fractions and 3′,4′,7-trihydroxyflavone from *Myristica fragrans* Houtt against MDR Gram-negative enteric bacteria

**DOI:** 10.1186/s12906-018-2084-1

**Published:** 2018-01-15

**Authors:** Joachim K. Dzotam, Ingrid Konga Simo, Gabin Bitchagno, Ilhami Celik, Louis P. Sandjo, Pierre Tane, Victor Kuete

**Affiliations:** 1 Department of Biochemistry, Faculty of Science, University of Dschang, P.O. Box 67, Dschang, Cameroon; 2 Department of Chemistry, Faculty of Science, University of Dschang, Dschang, Cameroon; 3 Department of Pharmaceutical Sciences, CCS, Universidade Federal de Santa Catarina, Florianópolis, Santa Catarina 88040-900 Brazil; 4 Department of Chemistry, Faculty of Science, Anadolu University, Eskişehir, Turkey

**Keywords:** 3′,4′,7-trihydroxyflavone, Antibiotic modifying activity, Cameroon, Gram-negative bacteria, Multidrug-resistance, *Myristica fragrans*

## Abstract

**Background:**

Nutmeg is the seed kernel inside the fruit of *Myristica fragrans* Houtt. (Myristicaceae). It possesses various pharmacological activities but is used in Cameroon only for its flavor in making cakes. The present study thus aimed to investigate the *in vitro* antibacterial activity and antibiotic modifying activities of crude seed kernel methanol extract (MFS), fractions (MFSa-e) as well as 3′,4′,7-trihydroxyflavone from *Myristica fragrans* against a panel of multi-drug resistant (MDR) Gram-negative bacteria.

**Methods:**

The modified rapid *p*-iodonitrotetrazolium chloride (INT) colorimetric assay was used to determine the Minimal Inhibitory Concentration (MIC) and Minimal Bactericidal Concentration (MBC) on the tested bacteria, as well as those of antibiotics in association with the extract and/or isolated compound. Column chromatography was used for the fractionation and purification of the seed kernel extract whilst the chemical structures of compounds were determined using spectroscopic techniques.

**Results:**

Phytochemical investigations lead to the isolation of 3′,4′,7-trihydroxyflavone from the fraction MFSb. The crude extract showed antibacterial activity with MICs ranging from 32 to 1024 μg/mL on the majority of the 29 tested Gram-negative bacterial strains. Fraction *MFSb* inhibited the growth of 100% (29/29) of the tested bacterial strains, as well as the compound 3′,4′,7-trihydroxyflavone (12/12) with a MIC values ranging from 32 to 1024 μg/mL, and 4 to 128 μg/mL respectively. The lowest MIC value (4 μg/mL) was recorded with 3′,4′,7-trihydroxyflavone against *Providencia stuartii* ATCC299645 as well as the best MBC value (16 μg/mL) against the same strain. In the presence of Phenylalanine-Arginine-β-Naphthylamide (PAßN), an efflux pumps inhibitor, the activity of the extract increased on 73.33% (11/15) meanwhile that of 3′,4′,7-trihydroxyflavone increased on 100% tested bacteria. The compound 3′,4′,7-trihydroxyflavone potentiated the activity of antibiotics in the majority of the tested bacterial strains.

**Conclusion:**

The results of the present work provide additional information on the use of nutmeg and it major antibacterial component, 3′,4′,7-trihydroxyflavone, as a potential drug in the treatment of bacterial infections including multidrug resistant phenotypes.

**Electronic supplementary material:**

The online version of this article (10.1186/s12906-018-2084-1) contains supplementary material, which is available to authorized users.

## Background

The use of antibiotics in human heath care or as a preventive measure in animal feed has greatly improved the living conditions of population and animal production. However, it has also gradually contributed to selection of resistant bacteria to different families of antibiotics [1]. Bacterial resistance is of considerable economic importance, and in combination with the undesirable side effects of some synthetic compounds, it becomes necessary and imperative to search for new and cheaper molecules with few side effects [2]. Plants and their derived products have long been used by humans for medicinal purposes. It is estimated today that about 80% of the world’s population uses botanical preparations as medicines to cover their health needs [3]. Besides, promissing new concepts such as efflux pump inhibitors [4] and synergy effect between antibiotics and plant secondary metabolites, are now well developed. Several bioactive spices against MDR Gram-negative bacteria, as well as their ability to potentiate the activity of commonly used antibiotics, have been recently reported. Some of these include: *Aframomum citratum* [5]; *Aframomum melegueta*, *Scorodophloeus zenkeri*, and *Tetrapleura tetraptera* [6]. In our continuous search for botanicals and phytochemicals to manage bacterial infections involving MDR Gram-negative bacteria, we targeted *Myristica fragrans* Houtt. (Myristicaceae) commonly known as nutmeg. Nutmeg is mostly used in Cameroon for its flavor in making cake. However, it is used traditionnaly in many other countries for several purposes, including: supporting digestion, relieving headache, stomach ache, insomnia, anti-malarial, aphrodisiac, anti-rheumatoid [7]. Ethanol extract of nutmeg seeds showed high anti-inflammatory effect [8], and more than 70% growth inhibition against human cancer cell line at a concentration of 100 μg/mL [9], as well as aphrodisiac effect [10]. Methanol extract from this spice caused cell death of jurkat leukemia T cell line by a mechanism involving SIRT 1 mRNA downregulation [11] and anti-caricinogenic activity [12]. Essential oil obtained from *Myristica fragrans* seeds has growth inhibition capability of bacterial spores and can be used as food preservative [13]. Previous phytochemical investigation of the plant led to the isolation of several compounds including: benzene derivatives (myristicin, elemicin, safrole) myristic acid, alpha-pinene, terpenes, beta-pinene and trimyristin [14, 15], trimyristin, derivatives of neolignans and eugenol [16] and also quercitin [8]. In the present study the bioguided fractionation was undertaken for depth investigation of the antibacterial activity, and antibiotic-modulating effect of methanol extract from *Mysristica fragrans* seeds.

## Methods

### General procedure

For compound characterization, ElectroSpray ionization mass spectrometry (ESI-MS), nuclear magnetic resonance (NMR) spectra, column chromatography (CC) and thin layer chromatography (TLC) were performed according to previously described protocols [17–19].

### Plant material and extraction

The dried seeds of *Myristica fragrans* were purchased in march 2015 from Douala central market, Littoral Region of Cameroon. The identification of plant (leaves, bark and seeds) was done at the National Herbarium (Yaounde, Cameroon) where a voucher specimens were deposited under the reference number 60342 HNC (YA). The powdered seeds of *M. frangrans* (1500 g) were macerated in methanol (MeOH, 5 L) for 48 h at room temperature. The extract was then concentrated under reduced pressure to give a semi-solid reddish brown fat residue, (370 g) which constituted the crude extract (MFS). The extract was then kept at 4 °C until further use.

#### Isolation and purification of bioactive compounds from the seeds extract of *M. fragrans*

Part of the crude extract (350 g) was subjected to silica gel column chromatography eluted with gradients of CH_2_Cl_2_-EtOAc then EtOAc-CH_3_OH. Seventy fractions of 400 mL were collected using mixtures of CH_2_Cl_2_-EtOAc 85:15, 70:30 and 30:70, evaporated under reduced pressure and gathered on the basis of their TLC profiles into five main fractions coded A-E (A: 1–6; B: 7–32; C: 33–51; D: 52–60; E: 61–70). These fractions were submitted first to antibacterial test against selected strains. For each bacterial species, a reference strain and at least one resistant strain were selected. In regards to the results obtained, fractions D (10 g) and E (5 g) were not further investigated due to their low activity. Fraction B (10 g) with the most considerable antibacterial activity was separated by a column chromatography over silica gel using a gradient of CH_2_Cl_2_-EtOAc (100:0, 95:5, 90:10, 85:15, 80:20, 75:25 and 70:30) to afford seven sub-fractions (FrB1-FrB7). Subfraction FrB4 was further purified over silica gel by isocratic CH_2_Cl_2_-EtOAc (2:98) to give 3′,4′,7-trihydroxyflavone (12 mg).

#### Antimicrobial assays

##### Chemicals for antimicrobial assay

The reference antibiotics tested in the present work were: chloramphenicol (CHL), tetracycline (TET), ciprofloxacin (CIP), streptomycin (STR), erythromycin (ERY) and kanamycin (KAN) obtained from Sigma-Aldrich (St. Quentin Fallavier, France). *p*-Iodonitrotetrazolium chloride ≥ 97% (INT, Sigma-Aldrich) was used as microbial growth indicator meanwhile phenylalanine-arginine-*β*-naphthylamide (PAßN) (Sigma-Aldrich) was used as efflux pump inhibitor (EPI).

##### Microbial strains and culture media

A total of 29 Gram-negative bacteria were investigated in this work and included reference (from American Type Culture Collection) and clinical (Laboratory collection) strains of *Escherichia coli*, *Enterobacter aerogenes*, *Klebsiella pneumoniae*, *Providencia stuartii* and *Pseudomonas aeruginosa*. Their resistance profiles were previously reported [20]. They were maintained on agar slant at 4 °C and subcultured on a fresh appropriate agar plates 24 h prior to any antimicrobial test. The activation of bacteria prior to any assay was done in Mueller Hinton Agar (Sigma) meanwhile antibacterial assays were carried out using Mueller Hinton broth (MHB; Sigma) [21].

##### Microbial susceptibility testing

Minimal inhibitory concentration (MIC) and minimal bactericidal concentration (MBC) of samples against the tested bacteria were determined by microplate dilution method using the rapid INT colorimetric assay according to previously described methods [22] with some modifications [21, 23, 24].

The role of efflux pumps in the susceptibility of Gram-negative bacteria was evaluated by testing the samples (crude extract and isolated compound) in the presence of an efflux pump inhibitor (EPI), the PAβN (at 30 μg/mL) against fifteen selected MDR phenotypes for the crude extract, and seven for the isolated compound.

To evaluate the potentiating effect of the tested crude extract and compound, a preliminary assay was performed using the association of extract or isolated compound at their various sub-inhibitory concentrations with antibiotics against one of a problematic bacteria, *P. aeruginosa* PA124. MIC/2 and MIC/4 of extract or isolated compound were selected as the best sub-inhibitory concentrations [6, 25] and were further used for samples-antibiotics combinations against the selected MDR microorganisms. For each sample-antibiotic association, the fractional inhibitory concentration (FIC) was determined as the ratio of MIC of Antibiotic in combination, versus MIC of Antibiotic alone (MIC_Antibiotic in combination_/MIC_Antibiotic alone_) and the interpretation made as follows: synergistic (≤ 0.5), indifferent (1 to 4), or antagonistic (> 4) [26, 27]. All experiments were done in triplicates.

## Results

### Isolated compound

**Fig. 1:**
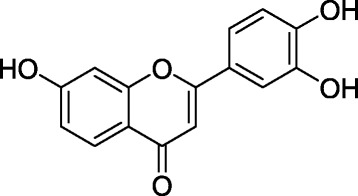
Chemical structure of 3′,4′,7-trihydroxyflavone isolated from the seeds of *Myristica fragrans* Houtt

The chemical structure of 3′,4′,7-trihydroxyflavone from the seeds of *M. fragrans* were elucidated using NMR (^1^H and ^13^C) data, in comparison with the literature [28] (Fig. 1). The ^1^H NMR, ^13^C NMR spectra and major chemical shifts of isolated compound are avalaible as Additional file 1. Extract, fractions, and 3′,4′,7-trihydroxyflavone identified in the seeds of *M. fragrans* were tested for their antimicrobial activities and antibiotic-modulating activity on a panel of Gram-negative bacteria. The results are reported in Tables 1, 2, 3 and 4.

**Table 1:** Minimal Inhibitory Concentration (MIC) in μg/mL of the crude MFS methanol extract, fractions and chloramphenicol

Bacterial strains		Tested samples, MIC and MBC, and MIC in the presence of PAßN in parenthesis (μg/mL)																				
		*MFS*			*MFSa*			*MFSb*			*MFSc*			*MFSd*			*MFSe*			*CHL*		
		*MIC*	*MBC*	*R*	*MIC*	*MBC*	*R*	*MIC*	*MBC*	*R*	*MIC*	*MBC*	*R*	*MIC*	*MBC*	*R*	*MIC*	*MBC*	*R*	*MIC*	*MBC*	*R*
*E. coli*	*ATCC8739*	**32**	512	16	256	1024	4	**32**	512	16	512	1024	2	1024	–	–	1024	–	–	4	16	4
	*ATCC10536*	**32**	1024	32	256	–	–	**32**	512	16	512	–	–	512	–	–	–	–	–	4	128	32
	*AG100*	**64**	1024	16	128	1024	8	**64**	1024	16	256	1024	4	512	–	–	–	–	–	8	64	8
	*AG100A*	128(32)	1024	8	256	256	1	**64**	64	1	256	256	1	512	512	1	–	–	–	64(4)	–	–
	*AG102*	**32(< 8)**	256	8	512	1024	2	**32**	512	16	128	1024	8	128	128	1	256	256	1	64(< 2)	256	4
	*AG100ATet*	256(16)	–	–	128	1024	8	**32**	128	4	512	1024	2	512	–	–	1024	–	–	128(4)	–	–
	*MC4100*	128	–	–	256	–	–	**64**	1024	16	512	–	–	512	–	–	512	–	–	8	64	8
	*W3110*	**32**	256	8	256	–	–	**64**	–	–	512	–	–	1024	–	–	–	–	–	8	256	32
*E. aerogenes*	*ATCC13048*	512	–	–	256	–	–	128	–	–	1024	–	–	1024	–	–	–	–	–	8	64	8
	*EA-CM64*	**64**(< 8)	–	–	128	512	4	**64**	512	8	512	1024	2	512	–	–	–	–	–	128(< 2)	–	–
	*EA3*	256	1024	4	256	–	–	**32**	1024	32	512	–	–	1024	–	–	–	–	–	32	256	8
	*EA27*	256(128)	–	–	128	1024	8	128	1024	8	256	1024	4	512	–	–	–	–	–	128(128)	–	–
	*EA289*	512(16)	–	–	512	–	–	256	–	–	512	–	–	512	–	–	–	–	–	256(16)	–	–
	*EA294*	**64**	512	8	128	1024	8	**32**	512	16	512	–	–	1024	–	–	–	–	–	8	128	16
	*EA298*	256(64)	1024	4	256	–	–	**64**	1024	16	1024	–	–	1024	–	–	–	–	–	32(16)	64	2
*K. pneumoniae*	*ATCC11296*	256	1024	4	128	–	–	**64**	512	8	512	1024	2	1024	–	–	–	–	–	64	–	–
	*K2*	1024	–	–	1024	1024	1	256	1024	4	1024	–	–	1024	–	–	–	–	–	32	128	4
	*K24*	**64**	512	8	256	1024	4	128	1024	8	1024	–	–	1024	–	–	–	–	–	128	256	2
	*KP55*	1024(512)	–	–	256	–	–	**64**	–	–	512	1024	2	512	–	–	–	–	–	64(32)	128	2
	*KP63*	512(64)	–	–	–	–	–	512	1024	2	–	–	–	–	–	–	–	–	–	64(32)	256	4
*P. stuartii*	*ATCC29916*	–	–	–	256	512	2	**32**	1024	32	512	1024	2	512	–	–	–	–	–	128	–	–
	*ATCC299645*	–	–	–	128	–	–	**64**	1024	16	512	–	–	1024	–	–	–	–	–	64	256	4
	*PS2636*	128(16)	256	2	256	–	–	**64**	512	8	512	1024	2	1024	1024	1	–	–	–	128(8)	–	–
	*NEA16*	128(16)	–	–	128	1024	8	**32**	1024	32	256	–	–	512	–	–	–	–	–	32(4)	256	8
*E. cloacae*	*BM47*	**64**(64)	1024	16	128	1024	8	**32**	128	4	512	–	–	1024	–	–	–	–	–	256(32)	–	–
	*BM67*	128(128)	–	–	–	–	–	512	–	–	1024	–	–	–	–	–	–	–	–	64(32)	–	–
	*ECCI69*	-(−)	–	–	–	–	–	512	–	–	1024	–	–	–	–	–	–	–	–	> 256(128)	256	–
*P. aeruginosa*	*PA01*	**64**	–	–	512	–	–	1024	–	–	1024	–	–	1024	–	–	1024	–	–	32	–	–
	*PA124*	1024(1024)	–	–	256	–	–	256	–	–	1024	–	–	1024	–	–	–	–	–	256(4)	–	–

R: CMB/CMI; −: values > 1024 for the extract and fractions, > 256 for chloramphenicol, or not calculated; (): values in parenthesis are MIC of substance in the presence of PAßN at 30 μg/mL; Values in Bold are the best MIC values for the crude MFS extract and its fractions

**Table 2:** Minimal Inhibitory Concentration (MIC) in μg/mL of isolated compounds and chloramphenicol

Bacterial strains		Tested samples, MIC and MBC and MIC in the presence ofPAßN in parenthesis (μg/mL)					
		3′,4′,7-Trihydroxyflavone			Chloramphénicol		
		*MIC*	*MBC*	*R*	*MIC*	*MBC*	*R*
*E. coli*	*ATCC8739*	32	256	8	4	16	4
	*AG102*	**8**(< 2)	128	16	16(< 2)	64	4
	*AG100ATet*	64(4)	128	2	128(4)	256	4
*E. aerogenes*	*ATCC13048*	64	–	–	8	64	4
	*EA27*	64(4)	–	–	128(128)	–	–
	*EA289*	64(32)	–	–	256(16)	–	–
*K. pneumoniae*	*ATCC11296*	32	128	4	16	128	8
	*KP63*	128(16)	–	–	64(32)	–	–
*P. stuartii*	*ATCC299645*	**4**	16	8	8	64	8
	*NEA16*	32(< 2)	128	4	32(4)	256	8
*P. aeruginosa*	*PA01*	64	–	–	–	–	–
	*PA124*	128(16)	256	2	–	–	–

R: CMB/CMI; −: values > 256 or not calculated; (): values in parenthesis are MIC of substance in the presence of PAßN at 30 μg/mL; Values in Bold are the best MIC values for the compounds

**Table 3:** MIC of different antibiotics after the association of the crude MFS extract at MIC/2, MIC/4 against ten MDR bacteria strains

Antibiotics	Bacterial strains, MIC (μg/mL) of antibiotics in the absence and presence of the extract											
	Extract concentration	PA124	AG102	AG100Atet	EA289	CM64	KP55	KP63	NEA16	PS2636	BM47	PBSS (%)
CHL	0	128	8	128	256	256	32	32	16	64	128	
	CMI/2	**32(0.25)** ^**S**^	**< 2(< 0.25)** ^**S**^	**64(0.5)** ^**S**^	256(1)^I^	**128(0.5)** ^**S**^	32(1)^I^	32(1)^I^	16(1)^I^	**16(0.25)** ^**S**^	128(1)^I^	**50**
	CMI/4	**32(0.25)** ^**S**^	**4(0.5)** ^**S**^	**64(0.5)** ^**S**^	256(1)^I^	**128(0.5)** ^**S**^	32(1)^I^	32(1)^I^	16(1)^I^	**16(0.25)** ^**S**^	128(1)^I^	**50**
CIP	0	64	2	> 64	> 64	> 64	2	< 0.5	4	8	< 0.5	
	CMI/2	**32(0.5)** ^**S**^	**< 0.5(< 0.25)** ^**S**^	> 64(≥1)^I^	**8(> 0.125)** ^**S**^	**32(> 0.5)** ^**S**^	2(1)^I^	< 0.5(≤1)^I^	**< 0.5(< 0.125)** ^**S**^	**< 0.5(< 0.062)** ^**S**^	< 0.5(≤1)^I^	**60**
	CMI/4	**32(0.5)** ^**S**^	**< 0.5(< 0.25)** ^**S**^	> 64(≥1)^I^	**16(> 0.25)** ^**S**^	**32(> 0.5)** ^**S**^	2(1)^I^	< 0.5(≤ 1)^I^	**< 0.5(< 0.125)** ^**S**^	**4(0.5)** ^**S**^	< 0.5(≤ 1)^I^	**60**
TET	0	16	16	32	64	4	32	2	4	2	32	
	CMI/2	32(2)^I^	**< 1(< 0.062)** ^**S**^	64(2)^I^	64(1)^I^	**< 0.5(< 0.125)** ^**S**^	32(1)^I^	**1(0.5)** ^**S**^	4(1)^I^	**< 1(< 0.5)** ^**S**^	32(1)^I^	40
	CMI/4	32(2)^I^	**< 1(< 0.062)** ^**S**^	64(2)^I^	64(1)^I^	**< 0.5(< 0.125)** ^**S**^	32(1)^I^	**1(0.5)** ^**S**^	4(1)^I^	**< 1(< 0.5)** ^**S**^	32(1)^I^	40
ERY	0	128	8	128	256	8	16	4	16	16	16	
	CMI/2	128(1)^I^	**< 2(< 0.25)** ^**S**^	128(1)^I^	256(1)^I^	**< 2(< 0,25)** ^**S**^	16(1)^I^	4(1)^I^	16(1)^I^	**8(0.5)** ^**S**^	16(1)^I^	30
	CMI/4	128(1)^I^	8(1)^I^	128(1)^I^	256(1)^I^	**< 2(< 0,25)** ^**S**^	16(1)^I^	4(1)^I^	16(1)^I^	16(1)^I^	16(1)^I^	10
KAN	0	128	4	8	32	32	32	< 2	16	16	8	
	CMI/2	128(1)^I^	**< 2(< 0.5)** ^**S**^	8(1)^I^	32(1)^I^	**16(0,5)** ^**S**^	32(1)^I^	< 2(≤ 1)^I^	**8(0.5)** ^**S**^	16(1)^I^	8(1)^I^	40
	CMI/4	128(1)^I^	**< 2(< 0.5)** ^**S**^	8(1)^I^	32(1)^I^	**16(0,5)** ^**S**^	32(1)^I^	< 2(≤ 1)^I^	**8(0.5)** ^**S**^	16(1)^I^	8(1)^I^	40
STR	0	256	> 256	> 256	128	8	64	8	16	> 256	64	
	CMI/2	**128(0.5)** ^**S**^	**128(≤ 0.5)** ^**S**^	**16(≤ 0.062)** ^**S**^	128(1)^I^	**< 2(< 0,25)** ^**S**^	64(1)^I^	**< 2(< 0.25)** ^**S**^	**8(0.5)** ^**S**^	**128(≤ 0.5)** ^**S**^	64(1)^I^	**70**
	CMI/4	**128(0.5)** ^**S**^	**256(≤ 1)** ^**S**^	**16(≤ 0.062)** ^**S**^	128(1)^I^	**< 2(< 0,25)** ^**S**^	64(1)^I^	8(1)^I^	**8(0.5)** ^**S**^	> 256(≥ 1)	64(1)^I^	**50**

^a^Antibotics [*TET* tetracycline, *CIP* ciprofloxacin, *STR* streptomycin, *KAN* kanamycin, *CHL* chloramphenicol, *ERY* erythromycin]

^b^Bacterial strains: *Escherichia coli* [AG102, AG100Atet], *Pseudomonas aeruginosa* [PA124], *Enterobacter aerogenes* [CM64, EA27, EA289], *Klebsiella pneumonia* [KP55, KP63], *Providencia stuartii* [NAE16], *Enterobacter cloacae* [BM47]

^c^*PBSS* percentage of bacteria strain on which synergism has been observed, (): *FIC* (Fractional Inhibitory Concentration) of the antibiotics after association with plant extract, *S* Synergy, *I* Indifference. The values in bold represent the cases of synergy between extract and antibiotic

**Table 4:** MIC of different antibiotics after the association with 3′,4′,7-Trihydroxyflavone MIC/2, MIC/4 against five MDR bacteria strains

Antibiotics	Bacterial strains, MIC (μg/mL) of antibiotics in the absence and presence of 3′,4′,7-Trihydroxyflavone						
	Compounds concentration	PA124	AG100Atet	EA289	NEA16	KP63	PBSS (%)
CHL	0	128	8	128	256	256	
	MIC/2	**16(0.125)** ^**S**^	**4(0.5)** ^**S**^	128(1)^I^	256(1)^I^	256(1)^I^	40
	MIC/4	**32(0.25)** ^**S**^	8(1)^I^	128(1)^I^	256(1)^I^	256(1)^I^	20
CIP	0	64	8	> 64	> 64	> 64	
	MIC/2	**32(0.5)** ^**S**^	**4(0.5)** ^**S**^	> 64(≥ 1)^I^	> 64(≥ 1)^I^	**32(˂0.5)** ^**S**^	60
	MIC/4	64(1)^I^	8(1)^I^	> 64(≥ 1)^I^	> 64(≥ 1)^I^	> 64(≥ 1)^I^	00
TET	0	16	16	32	64	4	
	MIC/2	**4(0.25)** ^**S**^	**< 1(< 0.062)** ^**S**^	64(2)^I^	**32(0.5)** ^**S**^	**< 0.5(< 0.125)** ^**S**^	**80**
	MIC/4	**8(0.5)** ^**S**^	**< 1(< 0.062)** ^**S**^	64(2)^I^	**32(0.5)** ^**S**^	**< 0.5(< 0.125)** ^**S**^	**80**
ERY	0	128	8	128	256	8	
	MIC/2	**16(0.125)** ^**S**^	**4(0.5)** ^**S**^	**16(0.5)** ^**S**^	**128(0.5)** ^**S**^	8(1)^I^	**80**
	MIC/4	**16(0.125)** ^**S**^	8(1)^I^	128(1)^I^	**128(0.5)** ^**S**^	8(1)^I^	40
KAN	0	128	4	8	32	32	
	MIC/2	**32(0.25)** ^**S**^	4(1)^I^	**4(0.5)** ^**S**^	32(1)^I^	**16(0.5)** ^**S**^	60
	MIC/4	**64(0.5)** ^**S**^	4(1)^I^	8(1)^I^	32(1)^I^	**16(0.5)** ^**S**^	40
STR	0	256	> 256	> 256	128	8	
	MIC/2	**16(0.0625)** ^**S**^	**256(< 1)** ^**S**^	> 256(≥ 1)^I^	128(1)^I^	**4(0.5)** ^**S**^	**60**
	MIC/4	**32(0.125)** ^**S**^	> 256(≥ 1)^I^	> 256(≥ 1)^I^	128(1)^I^	8(1)^I^	20

^a^Antibotics [*TET* tetracycline, *CIP* ciprofloxacin, *STR* streptomycin, *KAN* kanamycin, *CHL* chloramphenicol, *ERY* erythromycin]

^b^Bacterial strains: *Escherichia coli* [AG100Atet], *Pseudomonas aeruginosa* [PA124], *Enterobacter aerogenes* [EA289], *Klebsiella pneumonia* [KP63], *Providencia stuartii* [NAE16]

^c^*PBSS* percentage of bacteria strain on which synergism has been observed, (): FIC (Fractional Inhibitory Concentration) of the antibiotics after association with compounds, *S* Synergy, *I* Indifference; The values in bold represent the cases of synergy between extract and antibiotic

### Antibacterial activity

Crude seed extract (MFS), fractions MFSa-e and chloramphenicol were tested on a panel of 29 bacteria. The results summarized in Table 1 reveal selective activities with MIC values ranging from 32 to 1024 μg/mL for MFS against 26/29 (89.65%) tested bacteria as well as MFSb, MFSc, MFSa, MFSd and MFSe respectively against 29/29 (100%), 28/29 (96.55%), 26/29 (89.65%), 26/29 (89.65%) and 5/29 (13.79%) tested bacteria.

A keen look at data from Table 1 indicated that the MBC/MIC ratios were generally above 4. The antibacterial activities of 3′,4′,7-trihydroxyflavone compiled in Table 2 show that this compound inhibited the growth of all the 12 tested bacteria with MIC values ranging from 4 to 256 μg/mL.

### Role of efflux pumps in the susceptibility of gram-negative bacteria to the tested plant extracts

Fifteen of the studied MDR bacteria were also tested for their susceptibility to the crude plant extract (MFS), while seven were tested for their susceptibility to 3′,4′,7-trihydroxyflavone. This was done in the presence of PAβN at 30 μg/mL. The results showed that, when combined with the crude extract and isolated compounds, PAβN improves the activity (decrease of MIC values) of MFS, and 3′,4′,7-trihydroxyflavone on 11/15 (73.33%), and 7/7 (100%) of tested MDR strains respectively (Tables 1 and 2).

### Effects of the association of the extracts with antibiotics

A preliminary study performed against *P. aeruginosa* PA124, allowed us to choose the appropriate sub-inhibitory concentrations of MIC/2 and MIC/4 for further studies. The two samples were combined separately to six antibiotics (CIP, STR, CHL, ERY, KAN and TET) to evaluate their possible synergistic effects. The results summarized in Tables 3 and 4 showed that the synergistic effects were noted with all the tested samples with most of tested antibiotics. The activity of STR increased in the presence of almost all the tested samples at CMI/2,on at least 60% of the tested MDR. Also, the 3′,4′,7-trihydroxyflavone improved the activity of tetracycline and erythromycin on 80% of the tested bacteria, with FIC values ranging from 0.5 to < 0.062.

## Discussion

### Chemical composition

We isolated in the present study, a flavonoid (3′,4′,7-trihydroxyflavone) from the dried seeds of *M. fragrans*. Previous phytochemical studies on the same part of this plant revealed that the major chemical constituents are alkyl benzene derivatives (myristicin, elemicin, safrole), myristic acid, α-pinene, β-pinene and trimyristin [14, 15]. In the present work however, the isolation of a flavonoid from the methanol extract could probably be due to the fact that the purification was guided by the antibacterial activity and hence all fractions and sub-fractions were not explored.

### Antibacterial activity of the tested samples

According to established criteria [29, 30], the antibacterial activity of a plant extract is considered to be significant when the MIC values are below 100 μg/mL, moderate when 100 ≤ MIC ≤625 μg/mL and weak when MIC > 625 μg/mL. Consequently, the antibacterial activity of the crude seed extract (MFS) of *M. fragrans* could therefore be considered to be significant, since the MIC values below 100 μg/mL were obtained on the majority of the tested bacteria (Table 1). Fractionation, however yielded samples with sligtly the same activity as the crude extract, and the antibacterial effect could be considered to be moderate for most of them, but significant for the fractions MFSb (see Table 1). In fact, the lowest MIC value (32 μg/mL) was obtained with the crude MFS extract against 4 strains of *E. coli* (ATCC8739, ATCC10536, AG102 and W3110), as well as with the most active fraction MFSb against 9 bacteria strains (*E. coli*: ATCC8739, ATCC10536, AG102, AG100ATet; *E. aerogenes*: EA3, EA294; *P. stuartii*: ATCC29916, NEA16 and *E. cloacae* BM47). Importantly, the MIC values obtained with the crude MFS extract against *E. coli* AG102, *E. aerogenes* EA-CM64, *K. pneumoniae* K24 and *E. cloacae* BM47 (Table 1), and the most active fraction MFSb, against. *E. coli* AG102 and AG100ATet, *E. aerogenes* EA-CM64, *P. stuartii* ATCC29916 and PS2636, and *E. cloacae* BM47 (Table 1), were lower than those of chloramphenicol. It should be highlighted that MFSb had MIC values below 100 μg/mL against 19 of the 29 tested bacteria. The ratio MBC/MIC obtained were generally above 4, indicating that the studied extracts as well as the active fractions mostly exerted bacteriostatic effects [31]. Also, MIC and MBC values of the reference drug chloramphenicol were also very high (> 64 μg/mL) on several pathogens, confirming that most of the bacterial strains used were MDR phenotypes. The activity of compounds is significant when MIC< 10 μg/mL, moderate when 10 < MIC< 100 μg/mL and low when MIC > 100 μg/mL [29, 30]. On this basis, the activity of compound 3′,4′,7-trihydroxyflavone could be considered as significant against the strains of *E. coli* AG102 (MIC of 8 μg/mL) and *P. stuartii* ATCC299645 (MIC of 4 μg/mL) (Table 2).

### Role of efflux pumps in the susceptibility of gram-negative bacteria to the tested extracts

The MDR bacteria strains studied herein overexpressed efflux mechanism, which consist of expelling all toxic compounds (including antibiotics) out of their cytoplasm, preventing them from reaching their intracellular target [32]. In order to restore the intracellular concentration of antibacterials acting on intracellular target, efflux pumps could be blocked by an efflux pump inhibitor (EPI). In fact, in the presence of PAβN (EPI), the antibacterial activity of the crude MFS extract as well as that of 3′,4′,7-trihydroxyflavone has been improved on 11/15 (73,37%) and 7/7 (100%) respectively, on the tested MDR. These results suggested that the crude MFS extract as well as its active constituent, might be considered as substrates for efflux pumps, indicating that they may have an intracellular target [33].

### Effects of association of extracts with antibiotics

The loss of efficacy of commonly used antibiotics against MDR pathogens leads to the search of safety ways to improve, or at least to restore their activity. The combination of antibiotics with natural compounds is one of these strategies. In the present work, synergy was observed between the crude MFS extract, as well as its isolated compound with at least one of the six tested antibiotics against at least 50% of the MDR bacteria strains. A keen look from the results (Table 4) shows that 3′,4′,7-trihydroxyflavone has improved the activity of at least 70% of the tested antibiotics on more than 70% tested bacterial strains with FIC values, ranging mostly from 0.5 to < 0.0625. These results suggest that this compound could be considered as a potential efflux pump inhibitor [27].

## Conclusion

The present work demonstrated the important antibacterial activity of MFS crude extract and one of its derived compound, 3′,4′,7-trihydroxyflavone against MDR phenotypes. These two samples deserve further studies to develop new phytomedicines to manage bacterial infections involving MDR organisms.

## Additional file

Additional file 1: NMR data of 3′,4′,7-trihydroxyflavone. ^1^H NMR, ^13^C NMR spectra and chemical shifts of isolated compound. (DOCX 453 kb)

## Abbreviations

ATCC: American Type Culture Collection
CC: Column Chromatography
CFU: Colony forming unit
CHL: Chloramphenicol
CIP: Ciproflxacin
DMSO: Dimethyl sulfoxide
*E. aerogenes*: *Enterobacter aerogenes*
*E. cloacae*: *Enterobacter cloacae*
*E. coli*: *Escherichia coli*
ERY: Erythromycin
ESI-MS: ElectroSpray Ionization Mass Spectrometry
INT: *p*-iodonitrotetrazolium chloride
*K. pneumoniae*: *Klebsiella pneumoniae*
KAN: Kanamycin
MBC: Minimal bactericidal concentration
MDR: Multidrug resistant
MHB: Mueller Hinton Broth
MIC: Minimal inhibitory concentration
NHC: National Herbarium of Cameroon
NMR: Nuclear Magnetic Resonance
*P. aeruginosa*: *Pseudomonas aeruginosa*
*P. stuartii*: *Providencia stuartii*
RA: Reference antibiotic
SRPC: Scientific Research Projects Commission
STR: Streptomycin
TET: Tetracyclin
TLC: Thin Layer Chromatography
