# Supplementary material for: In vitro antibacterial and antibiotic modifying activity of crude extract, fractions and 3′,4′,7-trihydroxyflavone from Myristica fragrans Houtt against MDR Gram-negative enteric bacteria
Source: BMC Complement Altern Med. 2018 Jan 15;18:15. doi: 10.1186/s12906-018-2084-1 (PMC5769358; doi:10.1186/s12906-018-2084-1)
Supplement: Additional file 1: — NMR data of 3′,4′,7-trihydroxyflavone. 1H NMR, 13C NMR spectra and chemical shifts of isolated compound. (DOCX 453 kb) [file 12906_2018_2084_MOESM1_ESM.docx]

***In vitro* antibacterial and antibiotic modifying activity of crude extract, fractions and 3’,4’,7-trihydroxyflavonefrom *Myristica fragrans* Houtt against MDR Gram-negative enteric bacteria.**

Joachim K. Dzotam^1^, Ingrid Konga Simo^2^, Gabin Bitchagno^2^, Ilhami Celik^4^, Louis P. Sandjo^3^, Pierre Tane^2^, Victor Kuete^1*^

*^1^Department of Biochemistry, Faculty of Science, University of Dschang, Cameroon;*

*^2^Department of Chemistry, Faculty of Science, University of Dschang, Cameroon;*

*^3^Department of Pharmaceutical Sciences, CCS, Universidade Federal de Santa Catarina, Florianópolis 88040-900, Santa Catarina, Brazil;*

^4^*Department of Chemistry, Faculty of Science, Anadolu University, Eskişehir, Turkey;*

**Corresponding author:**

**Tel : (237) 77 35 59 27; Fax: (237) 22 22 60 18. P.O. Box 67 Dschang, Cameroon; E-mail:* [*kuetevictor@yahoo.fr*](mailto:kuetevictor@yahoo.fr) *(Prof. Dr. Victor Kuete)*

**E-mails:**

*Joachim K. Dzotam:* [*kamgue_joachim@yahoo.fr*](mailto:kamgue_joachim@yahoo.fr)

*Ingrid S. Konga:* [*simoingrid@yahoo.fr*](mailto:simoingrid@yahoo.fr)

*Gabin Bitchagno:* [*gabin1256@gmail.com*](mailto:gabin1256@gmail.com)

*Louis P. Sandjo:* [*plsansjo@yahoo.fr*](mailto:plsansjo@yahoo.fr)

*Ilhami Celik:* [*icelik@gmail.com*](mailto:icelik@gmail.com)

*Pierre Tane:* [*ptane109@gmail.com*](mailto:ptane109@gmail.com)

*Victor Kuete:* [*kuetevictor@yahoo.fr*](mailto:kuetevictor@yahoo.fr)

**NMR data of 3’,4’,7-trihydroxyflavone**

^1^H NMR of 3’,4’,7-trihydroxyflavone (400 MHz, (CD_3_)_2_SO)

^13^C NMR spectrum of 3’,4’,7-trihydroxyflavone (100 MHz, (CD_3_)_2_SO)

***3’,4’,7-trihydroxyflavone***: yellow oil. LR-ESI-MS m/z 271.1 [M+H]^+^; ^13^C NMR (CD_3_)_2_SO_,_ 100MHz: 146.0, 98.4, 181.5, 113.6, 125.8, 112.9, 167.8, 166.6, 123.7, 124.6 116.0, 148.3, 145.9, 117.9 [1]

**Reference**

[1] Junior GMV, de M. Sousa CM, Cavalheiro AJ, Lago JHG, Chaves MH. Phenolic derivatives from fruits of *Dipteryx lacunifera* Ducke and evaluation of their antiradical activities. *Helv Chim Acta.*2008;91: 2159-2167.
